# Distinct mobility facets and their association with intrinsic capacity domains in (pre-)frail community-dwelling older adults: an application of the unified framework for measuring mobility

**DOI:** 10.1186/s12877-026-07371-4

**Published:** 2026-03-23

**Authors:** Christian Werner, Tim Fleiner, Corinna Nerz, Gisela Büchele, Vanessa Haug, Christian Grüneberg, Martina Schäufele, Kilian Rapp, Jürgen M. Bauer

**Affiliations:** 1 Geriatric Center, Medical Faculty Heidelberg, Heidelberg University, Heidelberg, Germany; 2 Institute for Geriatric Research, Ulm University Medical Center, Ulm, Germany; 3 Agaplesion Bethesda Clinic, Geriatric Center Ulm, Ulm, Germany; 4 Institute of Medical Engineering and Mechatronics, Ulm University of Applied Sciences, Ulm, Germany; 5 Department of Clinical Gerontology, Robert-Bosch-Hospital, Stuttgart, Germany; 6 Institute for Epidemiology and Medical Biometry, Ulm University, Ulm, Germany; 7 Department of Nursing, Midwifery and Therapeutic Sciences, Bochum University of Applied Sciences, Bochum, Germany; 8 Department of Social Work, Technical University of Applied Sciences Mannheim, Mannheim, Germany

**Keywords:** Frailty, Older adults, Geriatrics, Functional ability, Mobility limitation, Locomotion, Intrinsic capacity

## Abstract

**Background:**

Maintaining mobility is essential for healthy aging. A recently proposed unified framework conceptualizes mobility through three facets: actual mobility, perceived mobility, and locomotor capacity. Identifying determinants of each facet is important for understanding mobility and informing tailored mobility-focused interventions. This is particularly relevant for (pre-)frail older adults, who are especially vulnerable to mobility decline. The aim of this study was to examine associations of the three mobility facets with intrinsic capacity (IC) domains and environmental factors in this population.

**Methods:**

This cross-sectional study used baseline data from 385 (pre-)frail community-dwelling older participants (81.2 ± 5.9 years, 73.5% women) of the randomized controlled PromeTheus trial. Actual mobility was assessed with the Life-Space Assessment, perceived mobility with the function component of the Late-Life Function & Disability Instrument, and locomotor capacity with the Short Physical Performance Battery (lower extremity function). Other IC domains included vitality (fatigue, nutritional status, handgrip strength), psychological (concerns about falling, global affect, loneliness), sensory (sensory index based on specialist visits), and cognitive (global function) capacities. Environmental factors comprised living situation, social network, city size, and weather conditions. Bivariate analyses identified candidate variables for multivariable linear regression models, adjusted for demographic and clinical characteristics.

**Results:**

In the multivariable analyses, actual mobility was positively associated with lower extremity function, nutritional status, social network, and sunshine duration. Perceived mobility was positively associated with lower extremity function, handgrip strength, city size, and negatively with concerns about falling. Locomotor capacity was positively associated with handgrip strength, cognitive function, city size, and cohabitation, and negatively with concerns about falling.

**Conclusions:**

The study revealed shared and facet-specific associations of actual mobility, perceived mobility, and locomotor capacity with IC domains and environmental factors. These findings highlight the multifactorial nature of mobility and may inform mobility-focused interventions that consider both shared and facet-specific determinants in (pre-)frail community-dwelling older adults.

**Trial registration:**

German Clinical Trials Register (DRKS00024638); prospectively registered on March 11, 2021.

## Background

Maintaining the ability to be mobile is a prerequisite for independence, participation, and quality of life in old age [1–3], and is recognized as a critical component of healthy aging [4]. The World Health Organization (WHO) defines healthy aging as the ‘process of developing and maintaining the functional ability that enables well-being in older age’ [4]. Functional ability refers to ‘the health-related attributes that enable individuals to be and to do what they have reason to value’ [5]. One key domain of functional ability is the ability to be mobile, alongside the ability to meet basic needs, to learn, grow, and make decisions, to build and maintain relationships, and to contribute to society [4].

Functional ability is determined by an individual’s intrinsic capacity (IC; ‘the composite of all the physical and mental capacities that an individual can draw on’), as well as by the environment and the interaction between these two [4]. Locomotor capacity, broadly defined as an individual’s physical capacity to move from one place to another, is one of the five domains of IC, together with vitality, psychological well-being, sensory function, and cognition [4]. Within the WHO framework for healthy aging, mobility is addressed both as a domain of functional ability (“the ability to be mobile”) and as a domain of IC (“locomotor capacity”), highlighting its central role in successful aging.

A robust and evidence-based measurement of mobility is essential to identify individuals with or at risk of mobility limitations and potential loss of functional ability, to guide tailored interventions, and to monitor changes over time or in response to interventions. Recently, a unified framework for comprehensive mobility measurement grounded in the WHO conceptualization of healthy aging has been proposed, encompassing three distinct facets of mobility: (1) perceived mobility, (2) actual mobility, and (3) locomotor capacity for mobility [6]. Perceived mobility refers to self-reported measures of an individual’s ability or difficulty in performing mobility-related tasks (‘what can you do?’). Actual mobility includes both self-reported measures of the frequency and duration of mobility in home and community settings, as well as objective measures of real-world mobility obtained through sensor technologies like accelerometry or Global Position System (‘what do you do in daily life?’). Locomotor capacity for mobility refers to physical performance-based measures of mobility in standardized settings, such as tests of gait speed, balance, or strength (‘what can you do in a test?’) [6].

Systematic reviews commissioned by the WHO to inform on mobility measurements for healthy aging propose the use of the function component of the Late-Life Function and Disability Instrument (LLFDI) [7] to assess perceived mobility [8] and the University of Alabama at Birmingham Study of Aging Life-Space Assessment (LSA) [9] to assess actual mobility [10]. To assess locomotor capacity, the WHO Guidelines on Integrated Care for Older People [11] recommend the Short Physical Performance Battery (SPPB, [12]). Together, perceived mobility and actual mobility constitute ‘the ability to be mobile’ domain of functional ability, while locomotor capacity for mobility aligns the ‘locomotor capacity’ domain of IC [6].

Understanding the determinants of these specific mobility facets can help to identify factors that contribute to or hinder mobility, and pave the way for developing comprehensive, tailored, and patient-centered interventions to preserve or promote overall mobility and, ultimately, functional ability. Determinants of individual mobility facets have been widely studied in community-dwelling older adults. Actual and perceived mobility have both been associated with various IC domains, including locomotor (e.g., gait speed, lower extremity function, balance) [7, 13, 14], vitality (e.g., nutritional status, handgrip strength) [7, 13–15], psychological (e.g., depressive symptoms, concerns about falling [CaF]) [7, 13–15], sensory (e.g., hearing, vision acuity) [14–17], and/or cognitive capacity (e.g., global cognitive function, executive functioning) [9, 14], as well as sociodemographic (e.g., age, education) [7, 18] and environmental factors (e.g., social support, walkability, weather conditions) [7, 14, 19, 20]. Associations between locomotor capacity for mobility and these determinants have also been well documented (vitality [21–23], psychological [24, 25], sensory [26], and/or cognitive capacity [27, 28]; sociodemographic and environmental factors [15, 29, 30]).

Despite growing knowledge about mobility determinants in older adults, most evidence results from studies that examined individual mobility facets in isolation and were conducted in non-frail populations. To our knowledge, no study has comprehensively assessed determinants of all three distinct mobility facets—actual mobility, perceived mobility, and locomotor capacity—specifically in (pre-)frail community-dwelling older adults. Given that frailty itself is associated with lower levels across all mobility facets [31–33], (pre-)frail older adults may show specific patterns of associations with IC domains. Evaluating these relationships is crucial, as frailty may modify both the strength and nature of these associations. Addressing this gap can deepen the understanding of mobility limitations in this vulnerable population and support more precise, targeted intervention strategies.

Therefore, the aim of this study was to investigate associations of distinct mobility facets (actual mobility, perceived mobility, locomotor capacity) with IC domains and environmental factors in (pre-)frail community-dwelling older adults.

## Methods

### Study design and setting

This is a secondary, cross-sectional analysis of the baseline data from the PromeTheus study, a three-center randomized controlled trial to evaluate the effectiveness of a 12-month home-based multifactorial interdisciplinary intervention for preventing functional and mobility decline in community-dwelling (pre-)frail older adults (registered in the German Clinical Trials Register on March 11, 2021; DRKS00024638). A detailed trial protocol of the PromeTheus study has been published [34]. The study was conducted between May 2021 and November 2023 at three study sites (Heidelberg, Stuttgart, Ulm) in the federal state of Baden-Württemberg, Germany. Reporting of this study followed the Strengthening the Reporting of Observational Studies in Epidemiology (STROBE) guideline for cross-sectional studies [35].

### Study sample

Participants were recruited between May 2021 and November 2022 via general practitioners (GPs), who pre-screened potential participants for eligibility during a routine visit in the GP’s private practice and referred eligible persons for further screening to the study sites, and via “direct contact” to potential participants per flyers, articles in local magazines and newspapers, and personalized letters. Details on the recruitment strategies and screening procedures have been reported elsewhere [34, 36]. Eligibility criteria for this secondary analysis were based on those of the parent PromeTheus study. Inclusion criteria were age ≥ 70 years, Clinical Frailty Scale (CFS) = 4 (“very mild frailty”), 5 (“mildly frail”) or 6 points (“moderately frail”) [37], living at home or in assisted living, ability to walk ≥ 10 meter with or without walking aid, and insured with the largest health insurance company in the German federal state of Baden-Württemberg (‘Allgemeine Ortskrankenkasse [AOK] Baden-Württemberg’). Exclusion criteria were ability to walk ≥ 800 m without walking aid or breaks, cognitive impairment (Short Orientation-Memory-Concentration Test [SOMCT] > 10 pt. [38]), insufficient German language skills, insufficient visual acuity (inability to read study-relevant written materials), medical conditions (heart failure (New York Heart Association functional class III-IV), stroke within the last 6 months, Parkinson Disease (Hoehn & Yahr stage ≥ 3), cancer, if currently under treatment (e.g., chemotherapy, radiation) or in an advanced stage, severe lung disease requiring (intermittent) oxygen supply, or multiple sclerosis). Screening was performed by the GPs (medical conditions) in their private practice and by research staff in a telephone interview and at participants’ homes (SOMCT), respectively, before baseline assessment.

Sample size was based on the primary outcome of the PromeTheus study, which was the intervention effect on the LLFDI-FC after 12 months, yielding a total of 400 participants. Details on the sample size calculation have been reported in the trial protocol [34].

### Data collection

All interviews and testing procedures were consistently administered by research staff with extensive training in all aspects of assessments. All data were collected in-person at participants’ home, except for sociodemographic information (age, sex, education, living situation) obtained during telephone screening and residential characteristics (city size) and weather conditions derived from publicly available databases.

**Fig. 1:**
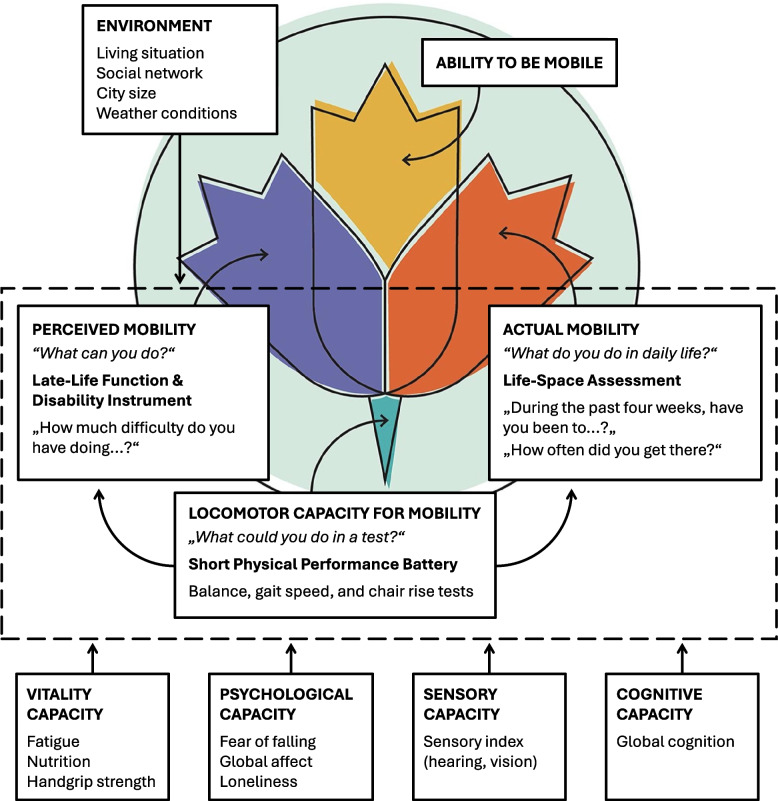
Overview of the unified framework for mobility measurement (modified from [6]), illustrating the three distinct mobility facets, IC domains, and environmental factors assessed in this study

An overview of the unified framework for comprehensive mobility measurement, including the distinct mobility facets, IC domains, and environmental factors assessed in this study, is presented in Fig. 1.

#### Perceived mobility

Perceived mobility was assessed using the function component of the Late-Life Function and Disability Instrument (LLFDI-FC) [7]. The LLFDI-FC evaluates self-reported difficulties in performing 32 physical activities on three functional domains: (1) upper extremity function (e.g. reaching behind the back), (2) basic lower extremity function (e.g. walking around) and advanced lower extremity function (e.g., getting up from the floor). Each item is rated from 1 (“none difficulty”) to 5 points (1 = “extreme difficulty/cannot do”). Individual item scores are transformed to create a LLFDI-FC summary score ranging from 0 to 100 points, with higher scores indicating higher physical functioning. A recent systematic review of the psychometric properties of measures of perceived mobility concludes that the LLFDI-FC can be used for this purpose in community-dwelling older adults [6].

#### Actual mobility

Actual mobility was assessed using the LSA [9], a self-reported instrument for evaluating life-space mobility. This assessment captures the spatial extent (outside the home, neighborhood, hometown, or beyond the hometown), frequency (< 1×/week, 1–3×/week, 4–6×/week, or daily), and level of independence (personal support, equipment, or without any support) of an individual’s mobility within the past 4 weeks. These dimensions are integrated into an LSA total score, which ranges from 0 (“totally bed-bound”) to 120 points (“daily mobility beyond the town without any support”). An LSA score of < 60 indicates restricted actual mobility [39]. The LSA has demonstrated adequate psychometric properties as a measure of actual real-world mobility in community-dwelling older adults [8].

#### Locomotor capacity for mobility

Locomotor capacity for mobility was assessed using the SPPB, including three tests of lower extremity function: a hierarchical standing balance test (Romberg, semi-tandem, and tandem stances), a usual gait speed test over 4 meter, and a 5-chair rise test [12]. Each test is scored with a maximum of 4 points, yielding a total score ranging from 0 to 12, with higher scores indicating greater locomotor capacity. A SPPB score of ≤ 9 points can be considered impaired locomotor capacity. The SPPB has been recommended by the WHO for the assessment of locomotor capacity [40].

#### Vitality capacity

Self-perceived fatigue was assessed using two items from the Center for Epidemiological Survey-Depression Scale: “I felt that everything I did was an effort” and “I could not get going during the past week” [41]. Fatigue was considered present if either question was answered with “a moderate amount of the time (3–4 days)” or “most or all of the time (5–7 days)”. Nutritional status was assessed using the Mini Nutritional Assessment-Short Form (MNA-SF), which includes six items on food intake, weight loss, mobility, acute disease, neuropsychological disorders, and Body Mass Index [42]. A higher MNA-SF score indicates a better nutritional status. The MNA-SF score can be categorized as follows: normal (≥ 12 points), at risk of malnutrition (≤ 11 points), and malnutrition (≤ 6 points). Handgrip strength (kg) was measured with a JAMAR digital hand dynamometer (sitting upright in a chair without armrest, elbow bent 90°, upper arm resting against the upper body). Two measurements were taken for each hand alternately, and the maximum value was recorded.

#### Psychological capacity

The 7-item Short Falls Efficacy Scale-International (Short FES-I) was used to evaluate CaF during physical and social activities of daily life [43]. Higher Short FES-I scores (range 7–28) indicate higher CaF. Short FES-I scores can be categorized as follows: low (7–8 pt.), moderate (9–13 pt.), and high (14–28 pt.) [44]. Global affect was measured with a visual analogue scale (VAS), ranging from 0 (worst) to 100 (best) [45]. Loneliness was assessed using the UCLA 3-item Loneliness Scale (UCLA-LS), which measures relational connectedness, social connectedness, and self-perceived isolation [46]. Higher UCLA-LS scores (range 3–9 pt.) indicate a higher level of perceived loneliness. A UCLA-LS score of ≥ 6 points indicate loneliness.

#### Sensory capacity

A sensory index was constructed based on items in the questionnaire for the use of medical and non-medical services in old age (FIMA; German: ‘*Fragebogen zur Inanspruchnahme medizinischer und nicht-medizinischer Versorgungsleistungen im Alter’*) [47]. The index incorporated information on visits to an ophthalmologist and/or an otorhinolaryngologist within the past six months, and the use of hearing aids as surrogates for sensory capacity. Scoring was defined as follows: use of a hearing aid = 2 points, ≥ 1 visit to an otorhinolaryngologist = 1 point, and ≥ 1 visit to an ophthalmologist = 1 point. The sensory index was calculated as the sum of these scores, with higher scores suggesting poorer sensory capacity.

#### Cognitive capacity

The SOMCT comprises six items on orientation (year, month, time), backward counting from 20, reciting months in reverse order, and recalling a name and address, with higher SOMCT scores (range 0–28 pt.) indicating lower cognitive capacity [38].

#### Environmental factors

Living situation (alone vs. with a person), social network (6-item Lubben Social Network Scale, LSNS-6) [48], city size (1 = rural municipality [< 5,000 inhabitants], 2 = very small town [5,000–9,999 inhabitants], 3 = small town [10,000–19,999 inhabitants], 4 = small medium-sized town [20,000–49,999 inhabitants], 5 = larger medium-sized town [50,000–99,999 inhabitants], 6 = small city [100,000–499,999 inhabitants], 7 = large city [≥ 500,000 inhabitants]), and weather conditions were assessed as environmental factors. Daily average temperature (°C), accumulated daily precipitation (mm), daily average humidity (%), accumulated daily sunshine duration (h), and rain days (n) were extracted for each participant from the weather station closest to their home address, sourced from the online agrometeorological databases of the German federal states of Baden-Württemberg and Bavaria [49, 50]. For the regression model on actual mobility (LSA), the data provided by the databases on a daily basis were averaged (temperature, humidity) or accumulated (precipitation, sunshine duration, rain days) over the four weeks prior to the assessment. For the regression model on perceived mobility (LLFDI-FC) and locomotor capacity (SPPB), weather data for the specific assessment day were extracted.

#### Covariables

Demographical and clinical characteristics included age, sex, education (years), number of medications, CFS [37], and fall history in the previous 6 months (yes vs. no).

### Statistical analysis

Descriptive data were presented as frequency (*n*) and percentage (%), median and interquartile range (IQR), or mean and standard deviation as appropriate. To identify potential predictors of actual mobility (LSA) and perceived mobility (LLFDI-FC), separate bivariate linear regression models were calculated for each measure of psychological (Short FES-I, VAS for global affect, UCLA-LS), vitality (self-perceived fatigue, MNA-SF, handgrip strength), sensory (sensory index), cognitive (SOMCT), and locomotor capacity (SPPB), as well as weather conditions (temperature, precipitation, humidity, sunshine duration, number of rain days). Candidate variables with a *p*-value < 0.20 [51] in the bivariate analysis were entered into a multivariable linear regression model (forced entry method), adjusted for demographic (age, sex, education) and clinical characteristics (number of medications, CFS, fall history) as potential confounders. Additionally, a linear regression model was constructed to examine the association of locomotor capacity (SPPB) with other IC domains and environmental factors, using the same bivariate and multivariable modeling approach. Analyses were performed using available data, and no imputation procedures were applied. For all multivariable models, linearity and homoscedasticity were confirmed through visual inspection of residual plots, and normality of residuals was supported by Q-Q plots. No multicollinearity was present among independent variables (tolerance > 0.1; variance inflation factor < 10). Unstandardized beta coefficients (B), standard errors (SE), standardized beta coefficients (*β*), *p*-values, and adjusted *R*^*2*^ statistics were reported for each model. Pearson correlation coefficients (*r*) were calculated to describe the bivariate associations between actual mobility, perceived mobility, and locomotor capacity. Potential sources of bias were addressed by using prespecified eligibility criteria from the parent PromeTheus trial, standardized assessments by trained research staff, and multivariable regression models adjusted for relevant confounders. Statistical analysis was performed using SPSS Statistics for Windows, Version 29.0 (IBM Corp., Armonk, NY, USA). Statistical significance was set exploratively at *p* < 0.05.

## Results

### Participant characteristics

The sample included 385 (pre-)frail community-dwelling older adults (age = 81.2 ± 5.9 years, females: *n* = 283, 73.5%). The median CFS score was 4 (“very mild frailty”) [IQR 4–5] points. More than one-third of participants (*n* = 142, 36.9%) experienced a fall within the last 6 months. Self-perceived fatigue was prevalent in 41% (*n* = 158), and malnutrition or risk of malnutrition in 19.4% (*n* = 75). More than 80% (*n* = 322) reported at least moderate fear of falling (Short FES-I ≥ 9 pt.). Approximately one-sixth *(n* = 65, 16.9%) were classified as being lonely (UCLA-LS ≥ 6 pt.). About one-third (*n* = 133, 34.5%) had a hearing aid. A mean SOMCT score of 4.6 ± 3.0 points indicated no to minimal cognitive impairment. About one-third each lived alone (*n* = 124, 32.2%) or were classified as socially isolated (LSNS-6 < 12 pt.: *n* = 128, 33.2%). Most participants (*n* = 274, 71.5%) resided in small or large cities (≥ 100,000 inhabitants). Further participant characteristics are presented in Table 1.

**Table 1:** Participant characteristics

Variable	*n* = 385^†^
Demographical & clinical characteristics	
Age, years	81.2 ± 5.9
Females, *n*	283 (73.5)
Education, years	11.3 ± 2.8
Medications, *n* (*n* = 383)	6.8 ± 3.5
CFS, pt.	4 [4–5]
Fall within the last 6 months, *n*	142 (36.9)
Locomotor capacity	
SPPB, pt.	6.5 ± 2.6
Impaired locomotor capacity (SPPB ≤ 9 pt.), *n*	295 (76.6)
Vitality capacity	
Fatigue, *n*	158 (41.0)
MNA-SF, pt.	12.7 ± 1.8
Nutritional status, *n*	
Normal (MNA-SF ≥ 12 pt.)	310 (80.5)
Risk of malnutrition (MNA-SF ≤ 11 pt.)	71 (18.4)
Malnutrition (MNA-SF ≤ 6 pt.)	4 (1.0)
Handgrip strength, kg	21.8 (8.9)
Psychosocial capacity	
Short FES-I, pt.	11 [9–14]
Concerns about falling,* n*	
Low (Short FES-I = 7–8 pt.)	63 (16.4)
Moderate (Short FES-I = 9–13 pt.)	187 (48.6)
High (Short FES-I = 14–28 pt.)	135 (35.1)
VAS global affect, pt. (*n* = 384)	67.7 ± 20.1
UCLA-LS, pt. (*n* = 384)	3 [1–4]
Lonely (UCLA-LS ≥ 6 pt.), *n* (*n* = 384)	65 (16.9)
Sensory capacity	
Ophthalmologist visit in the last 6 months, *n*	118 (30.6)
Otorhinolaryngologist visit in the last 6 months, *n*	213 (55.3)
Hearing aid, *n*	133 (34.5)
Sensory index (0 = best, 4 = worst)	1 [0–3]
Cognitive capacity	
SOMCT, pt.	4.6 ± 3.0
Environmental characteristics	
Living situation, *n*	
Alone	261 (67.8)
With a person	124 (32.2)
LSNS-6, pt. (*n* = 384)	13.9 ± 5.2
Social isolation (LSNS-6 < 12 pt.) (*n* = 384)	128 (33.3)
City size, *n*	
Rural municipality (< 5,000 inh.)	3 (0.8)
Very small town (5,000–9,999 inh.)	21 (5.5)
Small town (10,000–19,999 inh.)	50 (13.0)
Small medium-sized town (20,000–49,999 inh.)	22 (5.7)
Large medium-sized town (50,000–99,999 inh.)	15 (3.9)
Small city (100,000–499,999 inh.)	114 (29.9)
Large city (≥ 500,000 inh.)	160 (41.6)
Actual mobility	
LSA, pt	49.1 ± 18.7
Restricted mobility (LSA < 60 pt.), *n*	278 (72.2)
Perceived mobility	
LLFDI-FC, pt.	47.6 ± 7.7

Descriptive data given as *n* (%), median [interquartile range], or mean ± standard deviation

*CFS* Clinical Frailty Scale, *LSA* Life-Space Assessment, *LLFDI-FC* Late-Life Function and Disability Instrument, *LSNS-6* 6-item Lubben Social Network Scale, *MNA-SF* Mini-Nutritional Assessment–Short Form, *Short FES-I* Short Falls-Efficacy Scale-International, *SPPB* Short Physical Performance Battery, *UCLA-LS* UCLA 3-item loneliness scale, *VAS* visual analogue scale

^†^Unless otherwise indicated

### Description of mobility

Actual mobility was low, with a mean LSA score of 49.1 ± 18.7 points. A restricted actual mobility (LSA < 60 pt.) was observed in more than 70% (*n* = 278) (Table 1).

Perceived mobility was also limited, with a mean LLFDI-FC score of 47.6 ± 7.7 points.

Locomotor capacity for mobility was impaired, with 76.6% (*n* = 285) of participants scoring below 9 points in the SPPB, and the mean SPPB score averaged 6.5 ± 2.6 points. Mean gait speed was 0.68 ± 0.24 m/s, and the median time to complete the 5-chair rise test was 17.7 s [IQR 13.1–24.3]. The median SPPB balance score was 2 points [IQR 1–3]. Approximately one-sixth of the sample (*n* = 63; 16.4%) were unable to rise from a chair without using their hands (SPPB chair rise test score = 0 pt.).

Actual mobility showed moderate correlations with perceived mobility (LSA–LLFDI-FC: *r* = 0.544, *p* < 0.001) and locomotor capacity (LSA–SPPB; *r* = 0.562, *p* < 0.001). Locomotor capacity showed a strong correlation with perceived mobility (SPPB–LLFDI-FC: *r* = 0.702, *p* < 0.001).

### Predictors of mobility

Higher actual mobility (LSA) was independently associated with better nutritional status (*β* = 0.098, *p* = 0.018) (vitality capacity), greater lower extremity function (*β* = 0.312, *p* < 0.001) (locomotor capacity); a stronger social network (*β* = 0.091, *p* = 0.024) and longer sunshine duration (*β* = 0.087, *p* = 0.030) (environmental factors) (Table 2). This model explained 42.5% of the variance in actual mobility (adjusted *R*^2^ = 0.425).

**Table 2:** Bivariate and multivariable linear regression analyses of the associations between actual mobility (LSA) and IC domains and environmental factors

Variable	Bivariate analysis				Multivariable analysis^†^			
	***B***	**SE**	***β***	***p***	***B***	**SE**	***β***	***p***
Locomotor capacity								
Lower extremity function (SPPB)	4.016	0.302	0.562	**< 0.001**	2.229	0.376	0.312	**< 0.001**
Psychological capacity								
Concerns about falling (Short FES-I)	−1.566	0.204	−0.364	**< 0.001**	−0.136	0.209	−0.032	0.517
Global affect (VAS)	0.147	0.047	0.158	**0.002**	−0.018	0.041	−0.019	0.664
Loneliness (UCLA-LS)	−1.204	0.405	−0.150	**0.003**	−0.330	0.342	−0.041	0.335
Vitality capacity								
Self-perceived fatigue (0 = no, 1 = yes)	−7.014	1.913	−0.184	**< 0.001**	−0.464	1.702	−0.012	0.785
Nutritional status (MNA-SF)	1.907	0.519	0.184	**< 0.001**	1.018	0.428	0.098	**0.018**
Handgrip strength	0.467	0.105	0.222	**< 0.001**	−0.036	0.114	−0.035	0.756
Sensory capacity								
Sensory index (0 = best, 4 = worst)	−0.114	0.724	−0.008	0.875				
Cognitive capacity								
Cognitive function (SOMCT)	−1.286	0.310	−0.208	**< 0.001**	−0.220	0.263	−0.035	0.402
Environmental factors								
Living situation (0 = alone, 1 = with a person)	1.661	2.044	0.041	0.417				
Social network (LSNS-6)	0.601	0.180	0.168	**< 0.001**	0.324	0.143	0.091	**0.024**
City size	0.637	0.573	0.057	0.267				
Weather conditions^‡^								
Temperature	−0.152	0.160	−0.049	0.343				
Precipitation	−0.002	0.026	−0.005	0.926				
Humidity	0.005	0.063	0.004	0.930				
Sunshine duration	0.027	0.016	0.085	0.096	0.028	0.013	0.087	**0.030**
Rain days	0.241	1.921	0.006	0.900				

Bold *p*-values indicate statistical significance (< 0.05)

*Abbreviations*: *LSA* Life-Space Assessment, *LSNS-6* 6-item Lubben Social Network Scale, *MNA-SF* Mini-Nutritional Assessment-Short Form, *SOMCT* Short Orientation-Memory-Concentration Test, *SPPB* Short Physical Performance Battery, *Short FES-I* Short Falls Efficacy Scale-International, *UCLA-LS* UCLA 3-item loneliness scale, *VAS* visual analogue scale

^†^Adjusted for age, sex, education, medications, Clinical Frailty Scale, and fall history

^‡^Weather conditions were extracted over the four weeks prior to the assessment session

Higher perceived mobility (LLFDI-FC) was independently associated with lower CaF (*β* = –0.241, *p* < 0.001) (psychological capacity), higher handgrip strength (*β* = 0.098, *p* = 0.011) (vitality capacity); higher lower extremity function (*β* = 0.316, *p* < 0.001) (locomotor capacity); and larger city size (*β* = 0.095, *p* < 0.001) (environmental factors) (Table 3). This model explained 70.8% of the variance in perceived mobility (adjusted *R*^*2*^ = 0.708).

**Table 3:** Bivariate and multivariable linear regression analyses of the associations between perceived mobility (LLFDI-FC) and IC domains and environmental factors

Variable	**Bivariate analysis**				**Multivariable analysis**^†^			
	***B***	**SE**	***β***	***p***	***B***	**SE**	***β***	***p***
Locomotor capacity								
Lower extremity function (SPPB)	2.063	0.107	0.702	**< 0.001**	0.927	0.112	0.316	**< 0.001**
Psychological capacity								
Concerns about falling (Short FES-I)	−1.069	0.072	−0.607	**< 0.001**	−0.425	0.061	−0.241	**<.001**
Global affect (VAS)	0.102	0.019	0.266	**< 0.001**	0.013	0.012	0.035	0.258
Loneliness (UCLA-LS)	−0.764	0.271	−0.143	**0.005**	0.105	0.158	0.020	0.506
Vitality capacity								
Fatigue (0 = no, 1 = yes)	−5.046	0.758	−0.322	**< 0.001**	−0.705	0.499	−0.045	0.158
Nutritional status (MNA-SF)	0.637	0.215	0.150	**0.003**	0.076	0.126	0.018	0.548
Handgrip strength	0.312	0.041	0.359	**< 0.001**	0.085	0.033	0.098	**0.011**
Sensory capacity								
Sensory index (0 = best, 4 = worst)	−0.368	0.297	−0.063	0.216				
Cognitive capacity								
Cognitive function (SOMCT)	−0.368	0.129	−0.167	**0.001**	0.013	0.076	0.005	0.862
Environmental factors								
Living situation (0 = alone, 1 = with a person)	1.078	0.840	0.065	0.200				
Social network (LSNS-6)	0.086	0.075	0.059	0.252				
City size	0.869	0.232	0.188	**< 0.001**	0.437	0.134	0.095	**0.001**
Weather conditions^‡^								
Temperature	−0.102	0.061	−0.085	0.095	0.015	0.034	0.013	0.658
Precipitation	0.001	0.092	0.001	0.990				
Humidity	−0.003	0.026	−0.007	0.893				
Sunshine duration	0.018	0.095	0.009	0.854				
Rain day	−0.231	0.791	−0.015	0.771				

Bold *p*-values indicate statistical significance (< 0.05)

*Abbreviations*: *LLFDI-FC* Late-Life Function and Disability Instrument–Function Component, *LSA* Life-Space Assessment, *LSNS-6* 6-item Lubben Social Network Scale, *MNA-SF* Mini-Nutritional Assessment–Short Form, *SOMCT* Short Orientation-Memory-Concentration Test, *SPPB* Short Physical Performance Battery, *Short FES-I* Short Falls Efficacy Scale-International, *UCLA-LS* UCLA 3-item loneliness scale, *VAS* Visual Analogue Scale

^†^ Adjusted for age, sex, education, medications, Clinical Frailty Scale, and fall history

^‡^ Weather conditions were extracted for the specific assessment day

Higher locomotor capacity (SPPB) was independently associated with lower CaF (*β* = –0.288, *p* < 0.001) (psychological capacity); higher handgrip strength (*β* = 0.171, *p* < 0.001) (vitality capacity); higher cognitive function (i.e., lower SOMCT scores: *β* = –0.119, *p* = 0.003) (cognitive capacity); and living with another person (*β* = 0.104, *p* = 0.011) and in a larger city (*β* = 0.158, *p* = 0.001) (environmental factors) (Table 4). This model explained 46.6% of the variance in locomotor capacity (adjusted *R*^*2*^ = 0.466).

**Table 4:** Bivariate and multivariable linear regression analyses of the associations between locomotor capacity for mobility (SPPB) and other IC domains and environmental factors

Variable	**Bivariate analysis**				**Multivariable analysis**^†^			
	***B***	**SE**	***β***	***p***	***B***	**SE**	***β***	***p***
Psychological capacity								
Concerns about falling (Short FES-I)	−0.300	0.026	−0.501	**< 0.001**	−0.173	0.027	−0.288	**< 0.001**
Global affect (VAS)	0.023	0.007	0.177	**< 0.001**	0.000	0.005	0.002	0.968
Loneliness (UCLA-LS)	−0.219	0.092	−0.120	**0.018**	−0.003	0.073	−0.001	0.971
Vitality capacity								
Fatigue (0 = no, 1 = yes)	−1.105	0.267	−0.207	**< 0.001**	0.051	0.231	0.009	0.827
Nutritional status (MNA-SF)	0.186	0.073	0.128	**0.012**	0.078	0.058	0.054	0.180
Handgrip strength	0.095	0.014	0.323	**< 0.001**	0.051	0.015	0.171	**< 0.001**
Sensory capacity								
Sensory index (0 = best, 4 = worst)	0.014	0.101	0.007	0.888				
Cognitive capacity								
Cognitive function (SOMCT)	−0.217	0.043	−0.250	**< 0.001**	−0.104	0.035	−0.119	**0.003**
Environmental factors								
Living situation (0 = alone, 1 = with a person)	0.591	0.285	0.105	**0.039**	0.589	0.231	0.104	**0.011**
Social network (LSNS-6)	0.016	0.025	0.122	**0.016**	0.028	0.019	0.056	0.153
City size	0.299	0.079	0.190	**< 0.001**	0.248	0.061	0.158	**0.001**
Weather conditions^‡^								
Temperature	−0.043	0.021	−0.105	**0.039**	−0.010	0.016	−0.024	0.543
Precipitation	0.012	0.031	0.019	0.710				
Humidity	0.004	0.009	0.025	0.624				
Sunshine duration	0.032	0.032	0.051	0.320				
Rain day(s)	0.141	0.269	0.027	0.600				

Bold *p*-values indicate statistical significance (< 0.05)

*Abbreviations*: *LSNS-6* 6-item Lubben Social Network Scale, *MNA-SF* Mini-Nutritional Assessment–Short Form, *SOMCT* Short Orientation-Memory-Concentration Test, *SPPB* Short Physical Performance Battery, *Short FES-I* Falls Efficacy Scale-International, *UCLA-LS* UCLA 3-item loneliness scale

^†^Adjusted for age, sex, education, medications, Clinical Frailty Scale, and fall history

^‡^Weather conditions were extracted for the specific assessment day

## Discussion

This study examined associations between three distinct mobility facets—actual mobility, perceived mobility, and locomotor capacity—and IC domains and environmental factors in (pre-)frail community-dwelling older adults. To our knowledge, this is the first study to simultaneously investigate all three mobility facets within a single (pre-)frail sample, a population particularly vulnerable to mobility decline. The findings reveal both shared and facet-specific determinants across IC domains and environmental factors, indicating the multifactorial nature of mobility. Specifically, these findings suggest that mobility-focused interventions may need to distinguish between approaches targeting specific mobility facets and more comprehensive approaches addressing determinants relevant across multiple mobility facets.

### Actual mobility

Self-reported actual mobility was independently positively associated with locomotor capacity, vitality capacity (nutritional status), and environmental factors, including social network and sunshine duration. Among these, locomotor capacity showed the strongest association, which aligns with previous studies identifying it as the most powerful determinant of actual mobility in both higher-functioning community-dwelling older adults [9, 52] and those with cognitive impairment [53]. These findings highlight the importance of being able to move from one place to another as a prerequisite for interacting with one’s home and community environment.

The positive association with nutritional status, a key attribute of vitality capacity [54], is consistent with previous findings in older adults [13, 14, 52]. Adequate nutritional status may promote physical functioning, preserve muscle mass and energy levels [55], and thereby support engagement in mobility-related daily activities. In contrast, poor nutritional status may contribute to fatigue, muscle loss, and diminished physical resilience [56], thereby reducing motivation and ability for mobility in daily life.

The positive association with social network characteristics aligns with prior research demonstrating that greater social network size, support, and engagement are linked to higher levels of self-reported actual mobility in community-dwelling older adults [14, 52, 53, 57]. A broader and more integrated social network may increase opportunities or motivation for out-of-home activities. Being embedded in a closely connected social network may encourage regular mobility to maintain relationships, for example, through visiting friends or participating in social groups, thereby acting as both facilitator and driver of real-world mobility behavior.

The positive association with sunshine duration is consistent with previous findings indicating that weather conditions can influence actual mobility behavior in older adults [20, 58, 59]. Longer sunshine duration has been linked to increased self-reported time spent out-of-home and higher levels of objectively measured physical activity in community-dwelling older adults [20]. To our knowledge, this is the first study to demonstrate an independent association between sunshine duration and self-reported actual mobility among (pre-)frail older adults while accounting for a comprehensive range of IC domains and environmental factors. Previous studies typically relied on bivariate analyses or adjusted only for basic demographics [20, 58, 59]. These findings underscore the relevance of considering weather conditions when analyzing actual mobility in older adults.

Psychological capacity factors such as CaF and loneliness were not independently associated with actual mobility. This contrasts with findings from physically high-functioning older adults, in whom CaF has been identified as an independent predictor of self-reported actual mobility [60, 61]. However, studies in more physically impaired older adults have similarly found only bivariate associations between CaF and self-reported [53] or objectively measured actual mobility [62] that are attenuated after accounting for physical functioning, suggesting that this association may operate through locomotor capacity in such populations. Supporting this, CaF was independently associated with locomotor capacity in our study, reinforcing this mediating role. Evidence on the association between loneliness and actual mobility among older adults is mixed [63–66]. The absence of an independent association may reflect mediation through the social network size, consistent with findings showing independent effects of social isolation but not loneliness [65].

Cognitive capacity was also not independently associated with actual mobility. While a significant bivariate relationship was observed, it did not remain in the multivariable model, suggesting that its effect may be mediated by other factors such as locomotor or vitality capacity. This is consistent with previous findings showing that global cognitive status was no longer independently associated with actual mobility after adjusting for physical functioning in older adults [18]. The absence of an independent association may also reflect the limited cognitive variability in our sample, as individuals with cognitive impairment were excluded. In contrast, previous studies in more cognitively heterogeneous older populations have reported small but significant independent associations with global cognition [9] and executive functioning but not memory [14].

### Perceived mobility

Perceived mobility was independently positively associated with locomotor capacity, psychological capacity (CaF), vitality capacity (handgrip strength), and city size as an environmental factor.

The positive association with locomotor capacity aligns with findings in older adults with preclinical disability [67] or mobility impairments [68], supporting the view that perceived mobility is shaped, at least in part, by what individuals are physically capable of doing.

The negative association with CaF is consistent with findings showing that lower fall-related self-efficacy is linked to reduced perceived mobility [69, 70] and a higher likelihood of developing mobility disability [71], highlighting the role of psychological barriers in limiting self-perceived ability to perform mobility-related tasks.

The positive association with handgrip strength, a key indicator of vitality and overall muscle strength [4], suggests that physical robustness, beyond lower-extremity function, as captured by the SPPB (locomotor capacity), may also shape perceived mobility. Supporting this, previous studies have shown that lower neuromuscular function (handgrip or leg strength) is associated with greater likelihood of self-reported mobility limitations in older adults [72, 73].

Interestingly, living in larger urban areas was associated with higher perceived mobility. This may relate to community-based activities such as walking several blocks, climbing stairs, or getting in and out of vehicles, as assessed in the LLFDI-FC. Urban environments may support individuals’ perception of their ability to perform such tasks through better infrastructure, more frequent public transport, and mobility-promoting environmental cues. Although city size has not previously been identified as a direct predictor of perceived mobility, prior studies have shown that neighborhood and outdoor environmental features (e.g., poor lighting, lack of resting places, heavy traffic) can increase the risk of self-reported mobility limitations in older adults [74–76]. Together, these findings highlight the role of environmental context in shaping perceived mobility beyond individual capacity.

Cognitive capacity was not independently associated with perceived mobility, possibly because its effect may be mediated through other domains such as locomotor, psychological, or vitality capacity. As discussed above, the limited variability in cognitive function within our sample may also have contributed to the lack of association.

### Locomotor capacity

Locomotor capacity was independently associated with psychological capacity (CaF), vitality capacity (handgrip strength), cognitive capacity, and environmental factors including living with another person and in a larger city.

The negative association with CaF highlights the influence of psychological factors on objective physical function. This relationship is well-established among older adults [25, 77], suggesting a close interplay between physical and psychological components of mobility. CaF may contribute to more cautious movement strategies during performance-based mobility assessments and, over time, to activity restriction and physical deconditioning [78].

The positive association with handgrip strength underscores the interconnectedness of upper and lower extremity function as part of overall neuromuscular function and vitality capacity. This is consistent with prior research identifying handgrip strength as a useful marker of general physical capacity in older adults [23, 79].

The positive association with cognitive capacity reflects the well-known cognitive-motor interplay in aging. Lower cognitive function has been consistently linked to slower gait, poorer balance, reduced muscle strength, and weaker performance in mobility tasks among older adults [27, 28, 80, 81]. These findings underscore the importance of cognitive functioning in maintaining locomotor capacity for mobility in (pre-)frail older adults.

Living with another person was linked to better locomotor capacity, independent of the social network size. This is consistent with previous findings showing that living alone, also independent of other social contacts or engagement, is linked to lower locomotor capacity in older male hip fracture patients [82] and a greater risk of decline over time in community-dwelling older adults [83]. Cohabitation may support better locomotor capacity by promoting healthier behaviors (e.g., reduced sedentary time, better nutrition), facilitating engagement in mobility-related daily tasks (e.g., household chores, running errands), and increasing the likelihood that emerging health issues are noticed and addressed in a timely manner. These factors may help preserve physical capacity beyond what is provided by non-cohabiting social contacts.

The positive association with city size is consistent with previous findings suggesting that older adults in urban environments tend to have better locomotor capacity than those in rural areas [84]. Additionally, environmental attributes such as higher population density, greater availability of destinations, and better access to recreational facilities have been associated with better locomotor capacity in older adults [30, 85, 86]. Larger cities may offer better infrastructure for safe ambulation, more opportunities for incidental physical activity, and greater access to healthcare services, all of which may help maintain locomotor capacity. This complements the earlier findings for perceived mobility and further underscores the importance of the residential environment in shaping both subjective and objective aspects of mobility.

Sensory capacity has been previously identified to be associated with actual mobility [14], perceived mobility [16, 17], and locomotor capacity [26] in older adults. In our study, however, no such associations were observed across any of the three distinct mobility facets. This does not necessarily indicate a true absence of relationship but may reflect limitations in the operationalization of sensory capacity, relying on a surrogate index based on hearing aid use and recent specialist visits, as well as reduced variability due to the exclusion of individuals with insufficient visual acuity. Together, these factors likely limited the sensitivity of our measure and may have masked potential associations. Future research using specific assessments of sensory function is needed to better understand its association with the different mobility facets among (pre-)frail older adults.

Although actual mobility, perceived mobility, and locomotor capacity were examined in separate regression models, the present findings indicate that these mobility facets are closely related. Locomotor capacity showed the strongest independent association with both actual and perceived mobility, and actual and perceived mobility were moderately correlated with each other. This suggests that locomotor capacity represents a fundamental basis for both how mobility is enacted in the real-world environment and how abilities and difficulties in performing mobility-related daily tasks are perceived.

### Limitations

This study has several limitations. First, this was a secondary analysis of baseline data from the PromeTheus study not originally designed to comprehensively assess all IC domains. While locomotor, vitality, psychological, and cognitive capacities were measured using well-established and recommended outcome measures, sensory capacity was assessed using a surrogate index rather than specific functional assessments, which may have limited sensitivity. Second, due to the cross-sectional design, causal relationships between IC domains, environmental factors, and the three mobility facets cannot be inferred. Longitudinal studies are needed to clarify temporal relationships and directionality of these associations. Third, actual mobility was assessed using the LSA, a self-reported measure suggested within the unified framework for mobility measurement [6]. While the LSA is widely accepted and supported by well-established psychometric properties [10, 87], self-reported data may be influenced by recall bias or subjective interpretation. In future studies, real-world digital mobility outcomes derived from sensor-based technologies [88] may complement self-reported actual mobility and provide more objective insight into real-world mobility behavior. Fourth, the residential environment was assessed using city size only, which may not fully capture other environmental characteristics relevant to mobility, such as neighborhood walkability, availability of destinations, or access to public transportation. More detailed environmental assessments may help to better elucidate how such characteristics shape different mobility facets in future studies. Finally, participation was restricted to persons insured by AOK Baden-Württemberg, which may limit the generalizability of the findings to those insured by other statutory or private health insurance providers.

## Conclusion

This study demonstrates that actual mobility, perceived mobility, and locomotor capacity are associated with both shared and facet-specific determinants across IC domains and environmental factors in (pre-)frail older adults living in the community. Locomotor capacity emerged as the central determinant of both actual and perceived mobility, with vitality capacity showing additional associations with these mobility facets, while psychological capacity was specifically associated with perceived mobility. Locomotor capacity itself was related to psychological, vitality, and cognitive capacities. Environmental factors were relevant across all mobility facets but differed in their associations, with social and climatic factors linked to actual mobility, and residential factors linked to perceived mobility and locomotor capacity. These findings highlight the multifactorial nature of mobility and the importance of considering both shared and facet-specific determinants across different mobility facets.

## Data Availability

The datasets used and/or analyzed during the current study are available from the corresponding author on reasonable request.

## Abbreviations

CFS: Clinical Frailty Scale
GP: General practitioner
IC: Intrinsic capacity
LLFDI: Late-Life Function and Disability Instrument
LLFDI-FC: Late-Life Function and Disability Instrument-Function Component
LSA: Life-Space Assessment
LSNS-6: 6-Item Lubben Social Network Scale
MNA-SF: Mini-Nutritional Assessment-Short Form
SE: Standard error
Short FES-I: Short Falls Efficacy Scale-International
SOMCT: Short Orientation-Memory-Concentration Test
SPPB: Short Physical Performance Battery
UCLA-LS: UCLA 3-item Loneliness Scale
VAS: Visual analogue scale
WHO: World Health Organization
